# Profiling of cardio-metabolic risk factors and medication utilisation among Type II diabetes patients in Ghana: a prospective cohort study

**DOI:** 10.1186/s40169-017-0162-5

**Published:** 2017-09-07

**Authors:** Eric Adua, Peter Roberts, Samuel Asamoah Sakyi, Francis Agyemang Yeboah, Albert Dompreh, Kwasi Frimpong, Enoch Odame Anto, Wei Wang

**Affiliations:** 1 School of Medical and Health Sciences, Edith Cowan University, 270 Joondalup Drive, Joondalup, Perth, 6027 WA Australia; 2 Department of Molecular Medicine, Kwame Nkrumah University of Science and Technology, Kumasi, Ghana; 3 Department of Serology, Komfo Anokye Teaching Hospital, Kumasi, Ghana

**Keywords:** Type II diabetes mellitus, Hypertension, Anti-diabetic medications, Risk factors, Ghana

## Abstract

**Background:**

Type II diabetes mellitus (T2DM) is complicated by multiple cardio-metabolic risk factors. Controlling these factors requires lifestyle modifications alongside utilisation of anti-diabetic medications. Different glucose lowering [(biguanides (BIGs), sulfonylureas (SUAs), thiazolidinediones (TNZ)], lipid lowering (statins), and anti-hypertensive medicines [angiotensin converting enzyme inhibitors (ACEIs), calcium channel blockers (CCBs), angiotensin II receptor blockers (ARBs) and central acting drugs (CADs)] have been approved for controlling hyperglycaemia, dyslipidaemia and hypertension respectively. Here, we examined factors that characterise T2DM and explored the response to medication therapy among T2DM patients.

**Methods:**

This prospective cohort study recruited 241 T2DM patients reporting at a clinic in Ghana, from January through to August, 2016. Each patient’s demographic, medications and anthropometric data was obtained while information on medication adherence was captured using Morisky adherence scale-8 (MMAS-8). Fasting blood samples were collected for biochemical analysis.

**Results:**

The mean age of participants was 57.82 years for baseline and six-month follow-up. Physical activity differed at baseline and follow up (p < 0.05) but not body mass index (BMI). BIG alone, or in combination with SUA and TNZ did not improve glycaemic status at follow up (p > 0.05). Many participants using either ACEI or ARB were able to control their blood pressures. Among dyslipidaemia patients under statin treatment, there was an improved lipid profile at follow-up.

**Conclusions:**

Statin medications are effective for reducing dyslipidaemia in T2DM patients. However, control of modifiable risk factors, particularly blood glucose and to a lesser degree blood pressure is suboptimal. Addressing these will require concomitant interventions including education on medication adherence and correct dietary plans, lifestyle modifications and physical activity.

**Electronic supplementary material:**

The online version of this article (doi:10.1186/s40169-017-0162-5) contains supplementary material, which is available to authorized users.

## Background

Despite substantial efforts, type II diabetes mellitus (T2DM) remains a major contributor to the world’s morbidity and mortality [1, 2]. In 2014 alone, more than 2.2 million people died from the disease and at approximately the same time, nearly 415 million adults were affected worldwide, representing a prevalence rate of ≈8.5% [2, 3]. This prevalence rate is expected to translate into 439 million T2DM cases by 2030 [3, 4]. Unfortunately, countries with less healthcare resources such as those in sub-Saharan Africa (SSA) are among the most affected with some 14.2 million people presently suffering from the disease [2]. For example, in Ghana, T2DM affected more than 266,200 individuals at a prevalence rate of 6% in 2015, and it is presently ranked among the top 10 causes of all adult deaths [2, 5].

People with T2DM have an increased risk of developing many health problems such as cardiovascular diseases [6, 7], amputations [8], depression [9, 10], and cognitive impairment [11–14]. Moreover, prolonged hyperglycaemia is strongly linked with many microvascular and, to a lesser extent, macrovascular complications and premature mortality [15]. In fact, just a 1% rise in glucose level will lead to an 18% increased risk for cardiovascular events [16], 37% increased risk for renal diseases [11] and 12–14% increased risk for premature mortality [11, 15, 16].

Additionally, the majority of T2DM patients are physically inactive which has led to dyslipidaemia, obesity and hypertension [17, 18]. These in turn lead to further consequences. Studies have shown that obesity accounts for 14% of all adult deaths while hypertension alone is an independent risk factor for cognitive decline [19], renal dysfunction [20, 21] and ultimately responsible for 45% of all deaths. Therefore, given these detrimental outcomes, controlling known modifiable factors should be a priority.

It has long been documented that achieving good glycaemic levels is pivotal to delaying T2DM complications. According to the American Diabetes Association (ADA), reduction of microvascular and macrovascular complications is possible at HbA1c <7% [22]. This could be achieved with single, combination or multiple glucose lowering medications [23, 24].

Alongside maintaining normal glycaemic levels, therapeutic interventions should be extended to other concomitant factors such as dyslipidaemia, hypertension and obesity [25, 26]. Different lipid lowering and anti-hypertensive medicines have been approved for controlling dyslipidaemia and hypertension respectively; majority of which are currently available in Ghana [5, 26]. Yet, the control of T2DM modifiable factors has been suboptimal, partly because studies to create awareness of T2DM are generally scarce in this region. Moreover, these studies have mainly been cross-sectional providing limited information on association or causality. Therefore, in this study, we explored the manifestations and the associated factors that characterise T2DM in a longitudinal design. Additionally, this study highlights the proportion of T2DM patients that have good glycaemic control, blood pressure and lipid levels and addresses the factors that contribute to poor management and control of these modifiable risk factors.

## Methods

### Study design

This prospective cohort study was conducted at the diabetic clinic of the Komfo Anokye Teaching Hospital (KATH) from January through to August 2016. In all, 241 participants with T2DM aged 35–70 years who reported to the clinic for review and medications were recruited. The study protocol was reviewed by the Committee on Human Research, Publication and Ethics (CHRPE), Kwame Nkrumah University of Science and Technology (KNUST), Kumasi and the Human Research Ethics Committee (HREC), Edith Cowan University (ECU), Australia. Written informed consent was obtained from each participant.

### Inclusion and exclusion criteria

The study included only those who were diagnosed as having T2DM, based on the international classification of diabetes (ICD 10) criteria. Participants who were taking insulin injections were assumed to be suffering from type I diabetes mellitus and therefore were excluded. Additionally, among the original 260 T2DM participants recruited for the study, 19 were excluded, mainly because of missing clinical data.

### Anthropometric and blood pressure measurements

After obtaining demographic data and information on the general health status from each participant, information of medication adherence was obtained using the validated Morisky Adherence Scale-8 (MMAS-8). This questionnaire comprises 8 items and responses for item 1 through 7 are either ‘yes’ or ‘no’ whereas item 8 comprises a 5-point Likert scale [27]. Following this, anthropometric measurements were taken. Weight (kg) and height (cm) were measured with a standard stadiometer (SECA, Hamburg, Germany). These were used to determine the body mass index (BMI), calculated as BMI = weight (kg)/height (m)^2^. Waist and hip circumference were measured in cm using a tape measure and waist-to-hip ratio (WHR) was calculated as WHR = waist (cm)/hip (cm). Systolic blood pressure (SBP) and diastolic blood pressure (DBP) were measured using a standard sphygmomanometer (Omron HEM711DLX, UK). To assess the level of physical activity, we asked basic questions such as 1) what is the level of physical activity during the last 7 days?, 2) on how many days did you walk for at least 10 min at a time in your leisure time?

### Blood sample collection and biochemical assay

**Fig. 1:**
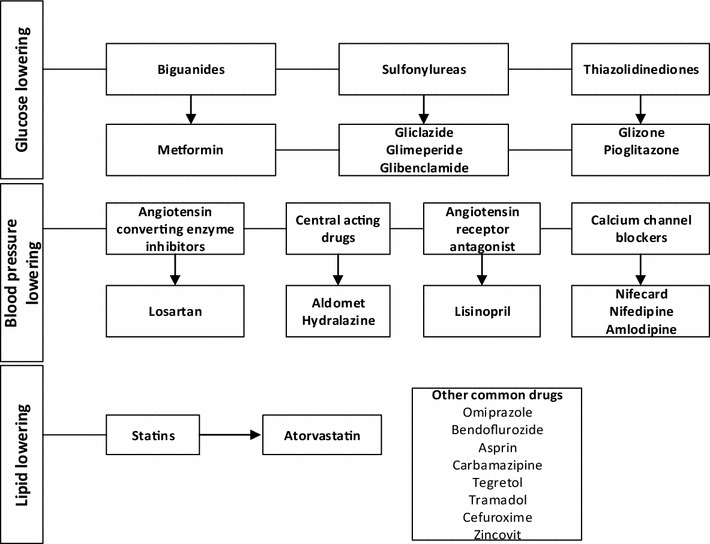
Category of medications utilised by T2DM patients

Venous fasting blood samples were collected from each participant into tubes containing EDTA (ethylene diamine tetraacetic acid), fluoride oxalate and gel separator. Fasting plasma glucose (FPG) in fluoride tubes and glycated haemoglobin (HbA1c) in EDTA tubes were measured on an automated chemistry analyser (Roche Diagnostics, COBAS INTEGRA 400 Plus, USA). Similarly, serum total cholesterol (TC), high density lipoprotein cholesterol (HDL-c), low density lipoprotein cholesterol (LDL-c), and triglycerides (TG) were measured on the automated chemistry analyser (Roche Diagnostics, COBAS INTEGRA 400 Plus, USA). Non-HDL was calculated as Non-HDL = total cholesterol-HDL. Coronary risk ratio and very low density lipoprotein (VLDL) cholesterol were calculated on the automated chemistry analyser. Various medications utilised by the T2DM patients at the clinic are shown in Fig. 1.

### Definition of terms

High plasma glucose; FBG >7 mmol/L, HbA1c >7.2% [28].

Normal BP; 140/90 mmHg, high SBP >140 mmHg, high DBP >90 mmHg [29].

Dyslipidaemia: waist circumference ≥102 cm (males), ≥88 cm (females), WHR >90 (men) and 0.85 (female). High TG ≥1.7 mmol/l, HDL-C <1.0 (male), 1.03 (female), high LDL-C ≥2.59 mmol/l, high total cholesterol ≥5.18 mmol/l, high non-HDL ≥3.37 mmol/l [30].

### Statistical analysis

Normality distribution was checked by the Shapiro–Wilk test. All continuous data was recorded as mean ± standard deviation and as frequency (percentages) for categorical variables. Between group comparisons for continuous variables were performed using student *t*-tests, and intergroup comparisons of categorical variables were performed using Chi square tests. Association between categorical variables and FBG or HbA1c were performed using logistic regression models and odds ratios (ORs) at 95% confidence intervals (95% CI) were recorded. All statistical analyses were performed using the Statistical Package for Social Sciences (SPSS), version 22. A *p* < *0.05* was considered significant.

## Results

Among the study population, the male to female ratio was 99/142 at baseline and 66/94 at follow up respectively. BMI and WHR of participants did not significantly differ from baseline to follow up [i.e. (p = 0.172) and (p = 0.276) respectively]. However, there was a significant difference in levels of physical activity from baseline to 6-month follow up (p = 0.0001) (Table 1).

**Table 1:** Socio-demographic characteristics of study participants: Baseline and follow up

Variable	Total	Baseline (n = 240)	Follow up (n = 160)	X^2^, df	*p* value
Age (years)	57.80 ± 10.63	57.82 ± 10.88	57.79 ± 10.39	0.370^t^	0.981
Male:female ratio	165/236	99/142	66/94		
BMI (Kg/m^2^)	26.80 ± 9.44	26.13 ± 5.11	27.47 ± 13.78	1.367^t^	0.172
WHR	0.93 ± 0.05	0.93 ± 0.06	0.92 ± 0.05	1.090^t^	0.276
Marital status				17.5, 3	*0.002*
Married	269 (67.1)	164 (68.0)	105 (65.6)		
Never married	6 (1.5)	4 (1.7)	2 (1.3)		
Divorced	41 (10.2)	25 (10.4)	16 (10.0)		
Widowed	85 (21.2)	48 (19.9)	37 (23.1)		
Education				3.01, 4	0.55
Tertiary	58 (14.5)	36 (14.9)	22 (13.8)		
Senior high school	104 (25.9)	57 (23.7)	47 (29.4)		
Junior high school	133 (33.2)	78 (32.4)	55 (34.4)		
Lower primary	43 (10.7)	28 (11.6)	15 (9.4)		
No formal education	63 (15.7)	42 (17.4)	21 (13.1)		
Occupation				39.65, 3	*0.0001*
Employed	229 (57.1)	133 (55.2)	96 (60.0)		
Retired	85 (21.2)	35 (14.5)	50 (31.1)		
Unemployed	65 (16.2)	51 (21.1)	14 (8.8)		
Informal employment	22 (5.5)	21 (9.0)	1 (1)		
Physical activity				25.22, 1	*0.0001*
Primarily sedentary	101 (25.2)	79 (32.8)	22 (13.8)		
Moderate activity	300 (74.6)	162 (67.2)	138 (85.5)		

Values are presented as frequency (percentage); mean ± SD

^t^t-test value

The majority of the study participants were aged 51–60 years [81 (33.6%) vs 55 (34.4%)] while the lowest age range was 31–40 years [14 (5.8%) vs 10 (6.3%)] (Table 2). The severity and mean levels of the measured parameters were not significantly different from baseline to follow-up; [SBP (p = 0.474 and p = 0.600), DBP (p = 0.382 and p = 0.620), FBG (p = 0.364 and p = 0.940), TC (p = 0.328 and p = 0.160), non-HDL (p = 0.270 and p = 0.250) and LDL-c (p = 0.092 and p = 0.430)]. However, there was a difference in the severity and mean levels of HbA1c [(p = 0.004 and p = 0.0001)], TG [(p = 0.006 and p = 0.0001)] and HDL-c [(p < 0.0001 and p = 0.0001)] from baseline to follow up (Table 2).

**Table 2:** Distribution of clinical characteristics among study participants

Variables	Total	Baseline (n = 241)	Follow-up (n = 160)	X^2^, df	p value
Age				0.909, 4	1.000
31–40	24 (6.0)	14 (5.8)	10 (6.3)		
41–50	76 (19.0)	49 (20.3)	27 (16.9)		
51–60	136 (33.9)	81 (33.6)	55 (34.4)		
61–70	118 (29.4)	68 (28.2)	50 (31.3)		
71–80	47 (11.7)	29 (12.0)	18 (11.3)		
BMI				3.386, 3	0.336
Underweight	11 (2.8)	9 (3.80)	2 (1.30)		
Normal weight	175 (43.9)	170 (44.6)	68 (42.8)		
Overweight	132 (33.10)	80 (33.3)	52 (32.7)		
Obese	81 (20.3)	44 (18.3)	37 (23.3)		
SBP				0.028, 1	0.474
Normal	121 (55.1)	132 (54.8)	89 (55.6)		
High	180 (44.9)	109 (45.2)	71 (44.4)		
DBP				0.178, 1	0.382
Normal	298 (74.5)	177 (73.8)	121 (75.6)		
High	102 (25.5)	63 (26.3)	39 (24.4)		
HbA1c				7.280, 1	0.004
Normal	104 (26.0)	74 (30.8)	30 (18.8)		
High	296 (74.0)	166 (69.2)	130 (81.3)		
FPG				0.202, 1	0.364
Normal	160 (39.9)	94 (39.0)	66 (41.3)		
High	241 (60.1)	147 (61.0)	94 (58.8)		
TG				6.679, 1	0.006
Good	343 (86.2)	199 (82.6)	144 (91.7)		
High	55 (13.8)	42 (17.4)	13 (8.3)		
TC				0.308, 1	0.328
Good	259 (65.2)	154 (64.2)	105 (66.9)		
High	138 (34.8)	86 (35.8)	52 (33.1)		
HDL				94.80, 1	*<0.0001*
Good	235 (59.0)	189 (78.4)	46 (29.3)		
Low	163 (41.0)	52 (21.6)	111 (70.7)		
NonHDL				0.474, 1	0.270
Normal	188 (47.4)	117 (48.8)	71 (45.2)		
High	209 (52.6)	123 (51.3)	86 (54.8)		
LDL				2.040, 1	0.092
Good	164 (41.3)	106 (44.2)	58 (36.9)		
High	233 (58.7)	134 (55.8)	99 (63.1)		
SBP (mmHg)	140.06 ± 24.09	139.41 ± 24.31	140.71 ± 23.88	0.525^t^	0.600
DBP (mmHg)	81.96 ± 13.18	81.63 ± 13.71	82.28 ± 12.65	0.484^t^	0.620
FBS (mmol/l)	18.32 ± 4.31	9.18 ± 4.42	9.14 ± 4.20	0.082^t^	0.940
HbA1c (mmol/l)	8.79 ± 2.49	8.27 ± 2.10	9.32 ± 2.88	4.201^t^	*0.0001*
TC (mmol/l)	4.63 ± 1.27	4.73 ± 1.27	4.54 ± 1.27	1.406^t^	0.160
TG (mmol/l)	1.17 ± 0.56	1.27 ± 0.57	1.07 ± 0.56	3.520^t^	*0.0001*
HDL-c (mmol/l)	1.19 ± 1.19	1.35 ± 1.35	1.03 ± 1.03	9.960^t^	*0.0001*
Non-HDL-c (mmol/l)	3.44 ± 1.22	3.37 ± 1.24	3.52 ± 1.20	1.142^t^	0.250
LDL-c (mmol/l)	2.91 ± 0.57	2.79 ± 1.16	3.03 ± 1.13	2.029^t^	0.430
Coronary risk	5.00 ± 2.7	4.97 ± 1.52	5.04 ± 3.88	0.232^t^	0.820
VLDL-c (mmol/l)	0.54 ± 0.33	0.58 ± 0.26	0.51 ± 0.41	1.965^t^	0.500

After adjusting for age and medication use, high BMI, SBP, DBP, TC, TG, HDL, non-HDL-c, and LDL-c status were not significant independent risk factors for high FBG in both baseline and follow up groups (p > 0.05) (Table 3). Similarly, in the logistic regression model, increased SBP, DBP, TC and non-HDL were slightly associated with high HbA1c levels at both baseline and follow up but not significantly (p > 0.05) (Table 4).

**Table 3:** Association between metabolic risk factors and FBG levels at baseline and follow up

Variables	FBG (baseline)		X^2^, df (p value)	p value	FBG (follow up)		X^2^, df (p value)	p value
	High (n = 147)	Normal (n = 94)	aOR (95% CI)		High (n = 94)	Normal (n = 66)	aOR (95% CI)	
Gender			1.38, 1 (0.239)				3.55, 1 (0.06)	
Male	56 (38.1)	43 (45.7)	1.0#		33 (35.1)	33 (50.0)	1.0#	
Female	91 (61.9)	51 (54.3)	1.37 (0.81–2.32)	0.283	61 (64.9)	33 (50.0)	1.85 (0.97–3.51)	0.073
BMI			2.18, 3 (0.537)				1.77, 3 (0.622)	
Underweight	6 (4.1)	3 (3.2)	1.05 (0.25–4.47)	1.000	2 (2.1)	1 (1.5)	1.40 (0.12–16.21)	1.000
Normal	70 (47.9)	37 (39.4)	1.0#		40 (42.6)	28 (43.1)	1.0#	
Overweight	46 (31.5)	34 (36.2)	0.72 (0.39–1.29)	0.289	29 (30.9)	23 (35.4)	0.88 (0.43–1.83)	0.852
Obese	24 (16.4)	20 (21.3)	0.63 (0.31–1.30)	0.268	23 (24.5)	14 (21.5)	1.15 (0.51–2.62)	0.836
SBP			0.87, 1 (0.351)				1.13, 1(0.288)	
Normal	77 (52.4)	55 (58.5)	1.0#		49 (52.1)	40 (60.6)	1.0#	
High	70 (47.6)	39 (41.5)	1.28 (0.76–2.16)	0.357	45 (47.9)	26 (39.4)	1.41 (0.75–2.68)	0.333
DBP			0.02, 1 (0.901)				2.34, 1(0.126)	
Normal	108 (73.5)	69 (74.2)	1.0#		67 (73.1)	54 (81.8)	1.0#	
High	39 (26.5)	24 (25.8)	1.04 (0.57–1.88)	1.000	27 (28.7)	12 (18.2)	1.81 (0.84–3.91)	0.139
TC			0.22, 1 (0.642)				0.09, 1 (0.764)	
Good	92 (63.0)	62 (66.0)	1.0#		62 (66.0)	43 (68.3)	1.0#	
High	54 (37.0)	32 (34.0)	1.14 (0.67–1.96)	0.681	32 (34.0)	20 (31.7)	1.11 (0.56–2.19)	0.863
TG			0.23, 1(0.630)				0.52,1 (0.472)	
Good	120 (81.6)	79 (84.0)	1.0#		85 (90.4)	59 (93.7)	1.0#	
High	27 (18.4)	15 (16.0)	1.19 (0.59–2.37)	0.729	9 (9.6)	4 (6.3)	1.56 (0.46–5.31)	0.565
HDL-c			0.01, 1(0.928)				0.83,1 (0.363)	
Good	115 (78.2)	74 (78.7)	1.0#		25 (26.6)	21 (33.3)	1.0#	
Low	32 (21.8)	20 (21.30)	1.03(0.55–1.93)	1.000	69 (73.4)	42 (66.7)	1.38 (0.69–2.77)	0.377
Non-HDL			0.05, 1 (0.827)				0.24,1 (0.621)	
Normal	72 (49.3)	45 (47.5)	1.0#		41 (43.6)	30 (47.6)	1.0#	
High	74 (50.7)	49 (52.1)	0.94 (0.56–1.59)	0.895	53 (56.4)	33 (52.4)	1.18 (0.62–2.23)	0.628
LDL-c			0.88, 1 (0.349)				0.84,1 (0.358)	
Good	68 (46.6)	38 (40.4)	1.0#		32 (34.0)	26 (41.3)	1.0#	
High	78 (53.4)	56 (59.6)	0.78 (0.46–1.32)	0.355	62 (66.0)	37 (58.7)	1.36 (0.71–2.63)	0.401

Logistic regression model, adjusted for age and medication. 1.0#: reference point for odds ratio

*X* ^*2*^, *df* Chi square value, degrees of freedom, *aOR* adjusted odds ratio, *CI* confidence interval

**Table 4:** Association between metabolic risk factors and HbA1c levels at baseline and follow up

Variables	HbA1c (baseline)		X^2^, df (p value)	p value	HbA1c (follow-up)		X^2^, df (p value)	p value
	Poor (n = 167)	Good (n = 74)	aOR (95% CI)		Poor (n = 130)	Good (n = 30)	aOR (95% CI)	
Gender			0.18, 1 (0.675)				0.96, 1 (0.328)	
Male	67 (40.4)	32 (43.2)	1.0#		56 (43.1)	10 (33.3)	1.0#	
Female	99 (59.6)	42 (56.8)	1.13 (0.65–1.96)	0.673	74 (56.9)	20 (66.7)	0.66 (0.29–1.52)	0.413
BMI			1.35, 3 (0.718)				4.38, 3 (0.224)	
Underweight	5 (3.0)	4 (5.4)	0.49 (0.12–1.94)	0.445	2 (1.6)	0 (0.0)		
Normal	77 (46.7)	30 (40.5)	1.0#		56 (43.4)	12 (40.0)	1.0#	
Overweight	53 (32.1)	26 (35.1)	0.79 (0.42–1.49)	0.519	45 (34.90	7 (23.3)	1.38 (0.50–3.78)	0.619
Obese	30 (18.2)	14 (18.9)	0.83 (0.39–1.79)	0.695	26 (20.2)	11 (36.7)	0.50 (0.19–1.29)	0.216
SBP			2.22,1 (0.136)				0.47, 1(0.491)	
Normal	86 (51.8)	46 (62.2)	1.0#		74 (56.9)	15 (50.0)	1.0#	
High SBP	80 (48.2)	28 (37.8)	1.53 (0.87–2.68)	0.161	56 (43.1)	15 (50.0)	0.76 (0.34–1.68)	0.544
DBP			0.16, 1 (0.692)				0.022, 1 (0.883)	
Normal	121 (72.9)	55 (75.3)	1.0#		98 (75.4)	23 (76.7)	1.0#	
High DBP	45 (27.1)	18 (24.7)	1.14 (0.60–2.14)	0.752	32 (24.6)	7 (23.3)	1.07 (0.42–2.73)	1.000
TC			2.42, 1 (0.12)				0.49, 1 (0.483)	
Good	101 (61.2)	53 (71.6)	1.0#		84 (65.6)	21 (72.4)	1.0#	
High	64 (38.8)	21 (28.4)	1.60 (0.88–2.89)	0.144	44 (34.4)	8 (27.6)	1.38 (0.56–3.36)	0.522
TG			0.00, 1 (0.985)				1.42, 1 (0.233)	
Good	137 (82.5)	61 (82.4)	1.0#		119 (93.0)	25 (86.2)	1.0#	
High	29 (17.5)	13 (17.6)	0.99 (0.48–2.04)	1.000	9 (7.0)	4 (13.8)	0.47 (0.14–1.68)	0.262
HDL-c			0.11, 1 (0.743)				0.46, 1 (0.499)	
Good	132 (79.0)	57 (77.0)	1.0#		39 (30.5)	7 (24.1)	1.0#	
Low	35 (21.0)	17 (23.0)	0.90 (0.46–1.73)	0.737	89 (69.5)	22 (75.9)	0.73 (0.29–1.84)	0.652
Non-HDL			0.25, 1 (0.620)				0.002, 1 (0.962)	
Normal	79 (47.9)	38 (51.4)	1.0#		58 (45.3)	13 (44.8)	1.0#	
High	86 (52.1)	36 (48.6)	1.15 (0.66–1.99)	0.675	70 (54.7)	16 (55.2)	0.98 (0.44–2.21)	1.000
LDL-c			0.05,1 (0.817)				0.53,1 (0.465)	
Good	74 (44.8)	32 (43.2)	1.0#		49 (38.3)	9 (31.0)	1.0#	
High	91 (55.2)	42 (56.8)	0.94 (0.54–1.630	0.888	79 (61.7)	20 (69.0)	0.73 (0.31–1.72)	0.528

Logistic regression model, adjusted for age and medication. 1.0#: reference point for odds ratio

*X* ^*2*^, *df* Chi square value, degrees of freedom, *aOR* adjusted odds ratio, *CI* confidence interval

From baseline to follow up, FBG levels increased by 25.0% when (BIG) was administered alone. In a combination therapy with either SUA or TNZ, there was only a decrease in FBG levels by 1% (p = 0.9924) and 1.6% (p = 0.1098) respectively. However, FBG levels decreased by 15.8% when all three medications; BIG, SUA and TNZ were administered (p = 0.216). Meanwhile, levels of HbA1c were increased by 29.6% after BIG treatment alone (p = 0.0094), increased by 19.2% and 16.7% when BIG was combined with SUA (p = 0.0175) and TNZ (p = 0.0903) respectively. However, a multiple therapy of BIG, SUA and TNZ resulted in only a 1.3% increase of HbA1c levels (p = 0.8308) (Table 5).

**Table 5:** Utilisation of glucose lowering medications among T2DM patients

	Baseline	Follow up	Mean difference (95% CI)	p value	% effect
Treatment					
FBG (mmol/l)					
BIG only	8.02 ± 0.65	10.08 ± 1.12	2.05 (−1.25 to 5.36)	0.2162	25.00
BIG + SUA	8.45 ± 0.49	8.441 ± 0.82	−0.01 (−1.83 to 1.82)	0.9924	−0.10
BIG + TNZ	9.63 ± 0.59	11.88 ± 1.47	2.25 (−0.52 to 5.02)	0.1098	23.40
BIG + SUA + TNZ	9.921 ± 0.66	8.36 ± 1.04	−1.57 (−4.06 to 0.93)	0.216	−15.80
HbA1c (%)					
BIG only	7.34 ± 0.28	9.51 ± 1.10	2.17 (0.57 to 3.78)	*0.0094*	29.60
BIG + SUA	8.11 ± 0.32	9.67 ± 0.65	1.55 (0.28 to 2.83)	*0.0175*	19.20
BIG + TNZ	8.68 ± 0.33	10.12 ± 1.04	1.45 (−0.23 to 3.14)	0.0903	16.70
BIG + Sul + TNZ	8.46 ± 0.26	8.57 ± 0.47	0.11 (−0.91 to 1.12)	0.8308	1.30

*BIG* Biguanide, *SUA* Sulfonylurea, *TNZ* Thiazolidinedione

p < 0.05 is considered significant

There was a mean percentage decrease effect in levels of HDL-c (p < 0.0001), TG (p = 0.0259) and VLDL-c (p = 0.0237) by 22.8%, 18.4% and 17.3% respectively, after atorvastatin treatment alone. Conversely, there was an increased effect in levels of TC (p = 0.743) by 1.7%, non-HDL-c (p = 0.075) by 14.5%, LDL-c (p = 0.022) by 21.5% and CR (p = 0.955) by 0.5% after atorvastatin treatment (Table 6).

**Table 6:** Utilisation of lipid lowering medications among T2DM patients

Variable	Baseline	Follow up	Mean difference (95% CI)	p value	% difference
TC (mmol/l)					
No statin	5.03 ± 0.12	4.95 ± 0.13	−0.07 (−0.42 to 0.28)	0.6817	1.39
Atorvastatin	4.06 ± 0.16	4.13 ± 0.16	0.07 (−0.37 to 0.51)	0.7434	1.72
TG (mmol/l)					
No statin	1.32 ± 0.06	1.19 ± 0.06	−0.13 (−0.30 to 0.04)	0.1322	9.85
Atorvastatin	1.14 ± 0.06	0.93 ± 0.06	−0.21 (−0.39 to −0.03)	*0.0259*	18.42
HDL-c (mmol/l)					
No statin	1.36 ± 0.03	1.05 ± 0.03	−0.30 (−0.39 to −0.21)	*<0.0001*	22.06
Atorvastatin	1.36 ± 0.04	1.04 ± 0.04	−0.31 (−0.42 to −0.19)	*<0.0001*	22.79
Non-HDL (mmol/l)					
No statin	3.67 ± 0.11	3.89 ± 0.12	0.23 (−0.09 to 0.55)	0.1617	6.27
Atorvastatin	2.69 ± 0.15	3.09 ± 0.15	0.39 (−0.04 to 0.81)	0.0754	14.50
LDL-c (mmol/l)					
No statin	3.06 ± 0.11	3.36 ± 0.11	0.30 (−0.01 to 0.60)	0.058	9.80
Atorvastatin	2.19 ± 0.15	2.68 ± 0.14	0.47 (0.06 to 0.87)	*0.022*	21.46
CR					
No statin	5.24 ± 0.15	5.57 ± 0.49	0.33 (−0.68 to 1.34)	0.5202	6.29
Atorvastatin	4.31 ± 0.19	4.32 ± 0.20	0.02 (−0.53 to 0.57)	0.9547	0.46
VLDL-c (mmol/l)					
No statin	0.60 ± 0.03	0.58 ± 0.05	−0.01 (−0.12 to 0.09)	0.8181	1.67
Atorvastatin	0.52 ± 0.03	0.42 ± 0.03	−0.09 (−0.18 to −0.01)	*0.0237*	17.3

*CI* confidence interval

p < 0.05 is considered significant

For non-hypertensive T2DM participants, there was no significant change in SBP and DBP from baseline to follow up (Table 7). SBP levels were reduced by 0.1% after CCB + ACEI treatment (p = 0.969). Levels of both SBP and DBP were reduced by 1.9% (p = 0.644) and 5.8% (p = 0.128) respectively after ACEI treatment alone and decreased by 1.0% (p = 0.835) and 0.1% (p = 0.912) respectively after CCB + ARB combination therapies. However, levels of both SBP and DBP increased by 3.0% (p = 0.683) and 0.4% (p = 0.942) respectively after CCB treatment alone and increased by 17.3% (p = 0.061) and 11.3% (p = 0.086) respectively after CAD treatment alone, while a combination therapy of CCB + ACEI increased DBP by 1.9% (p = 0.666) (Table 7).

**Table 7:** Utilisation of anti-hypertensive medicines among T2DM patients

Anti-hypertensive drugs	Baseline	Follow up	Mean difference (95%CI)	p value	%effect
	SBP (mmHg)				
DM only (n = 38)	127.1 ± 4.09	130.5 ± 3.45	3.34 (−7.33 to 14.01)	0.534	2.63
DM + HPT					
CCB (n = 11)	150.2 ± 7.99	154.6 ± 7.21	4.46 (−18.02 to 26.92)	0.683	2.96
ARB (n = 22)	130.1 ± 3.04	130.0 ± 4.36	0.01 (−10.74 to 10.74)	>0.999	0.00
ACEI (n = 30)	130.4 ± 3.93	128.0 ± 3.59	−2.47 (−13.12 to 8.19)	0.644	−1.90
CAD (n = 8)	150.6 ± 9.07	176.6 ± 8.33	26.0 (−2.43 to 54.43)	0.061	17.30
CCB + ARB (n = 24)	153.3 ± 5.74	151.7 ± 4.98	−1.58 (−16.88 to 13.72)	0.835	1.03
CCB + ACEI (n = 27)	143.1 ± 3.33	142.9 ± 3.46	−0.19 (−9.83 to 9.46)	0.969	0.13
	DBP (mmHg)				
DM only (n = 38)	74.87 ± 2.25	77.87 ± 1.80	3.00 (−2.75 to 8.74)	0.301	4.00
DM + HPT					
CCB (n = 11)	83.18 ± 3.74	83.55 ± 3.23	0.36 (−9.95 to 10.68)	0.942	0.43
ARB (n = 22)	80.00 ± 2.31	80.02 ± 2.09	1.00 (−5.29 to 7.30)	0.750	1.25
ACEI (n = 30)	80.01 ± 1.89	76.43 ± 2.37	−4.67 (−10.73 to 1.40)	0.128	5.76
CAD (n = 8)	93.80 ± 5.23	104.4 ± 7.22	10.6 (−9.96 to 31.16)	0.086	11.30
CCB + ARB (n = 24)	86.13 ± 3.33	86.08 ± 2.62	−0.04 (−8.58 to 8.50)	0.992	0.05
CCB + ACEI (n = 27)	82.81 ± 2.78	84.41 ± 2.40	1.59 (−5.78 to 8.96)	0.666	1.92

*CCB* calcium channel blockers, *ACEI* angiotensin converting enzyme inhibitors, *ARB* angiotensin II receptor blockers, *CAD*central acting drugs

## Discussion

The prevalence of T2DM has increased tremendously in the past few decades among different countries worldwide [2, 3, 31–34]. SSA remains one of the most affected regions due to rapid urbanisation and increased adoption of a westernised diet with less physical activity [5, 30–34].

In this hospital-based study, we examined the major factors that characterise T2DM and how these factors influence anti-diabetes medication response. As reported by Danquah et al. [5], the majority of T2DM patients in urban Ghana are middle aged, of low socio-economic status and their lifestyle is primarily sedentary [5]. Moreover, our findings on clinical parameters such as SBP, DBP, HDL-c, LDL-c, TG, TC and FBG are similar to those reported in their study [5].

Overall, several of these biomarkers are higher than the recommended threshold for T2DM as suggested by the WHO and the ADA [35, 36]. For example, approximately 60 and 69.2% of the participants were not able to achieve the desired FBG and HbA1c targets respectively. This is in fact disturbing given the direct association between abnormal plasma glucose levels and macrovascular or microvascular complications. Efforts to control glucose levels are necessary and could be achieved in several ways. After diagnosis, medical nutrition therapy (MNT) is necessary to reduce weight and normalise glucose levels [37].

However, it has been established that MNT alone is not sufficient for improving plasma glucose levels. As such, the use of medications becomes the next phase of action [37].

In Ghana, several glucose lowering medications have been approved for the treatment of hyperglycaemia including SUAs, TNZs and biguanides, the latter being the first line anti-diabetic medicine [5]. Like many other countries, its popularity is because: (1) it is less expensive, (2) it is effective for weight reduction and plasma glucose levels, and (3) it has a reduced risk for hypoglycaemia [38]. Not surprisingly, a high proportion of our participants (>80%) were on this medication, most of whom had used this drug for a period long before the start of this study. However, the majority of those who used BIG (metformin) alone could not achieve the desired glycaemic level even at follow up although there seems to be a minimal percentage effect (29.6%, p = 0.0094) on HbA1c level (Tables 3, 4, 5). This emphasises the failure of metformin as a monotherapy to achieve glucose control. At this point, the focus shifts towards individuals undergoing combination and multiple therapies.

SUAs and TNZs have been recognised as second line anti-diabetic medications and their efficacy is similar to metformin [25, 38]. However, it was apparent after six months that even with multiple therapies, the majority of the patients could not attain the desired glucose target levels. Only a minimal percentage effect of BIG + SUA (19.2%, p = 0.0175) on HbA1c was observed (Table 5). Several reasons can be attributed to this:

Firstly, there is a possibility of poor adherence to oral medications, especially among those taking combination and multiple therapies, not only for hyperglycaemia but also for other comorbidities [26, 38, 39]. Moreover, many of these drugs are associated with side effects and hence it is possible that some participants will be selective in their choice of medicine (Additional file 1: Table S1). In a study among 2849 T2DM patients in the UK, it was shown that only 13% of the patients adhered strictly to the drug regimen [40]. This could possibly be the case in our study as some participants may have become bored with swallowing different medications daily. Efforts to simplify treatment regimens should therefore be intensified. For example, instead of multiple medications, single-dose combination pills with minimal side effects could be administered. Secondly, ensuring adequate control of glycaemic status requires a paradigm shift from sedentary behaviour to a more physically active lifestyle. One study has shown that moderate-intensity physical activity such as brisk walking and reducing time spent watching television to less than 30 min per day could reduce several modifiable T2DM risk factors including plasma LDL-c and TG while increasing HDL-c [41]. A meta-analysis also showed that physical activity is inversely associated with risk for T2DM [42].

Moreover, intense exercise is necessary to stimulate 5-adenosine monophosphate-activated kinase (5-AMPK) causing the release of glucose to the muscles rather than it accumulating in the plasma [4]. In our study however, we were unable to assess the level or intensity of physical activity by the individuals. Therefore, an effective physical assessment tool such as the international physical activity questionnaire (IPAQ) could be useful [43].

Thirdly, poor dietary preferences may have been a contributory factor. Studies have shown that healthy diets or consumption of vegetables, low calorie diets, low trans fats, legumes, fruits, poultry, whole grains and cereal fibre is linked to a reduced risk of metabolic syndrome and T2DM [44, 45]. Conversely, consumption of red and processed meat, sugar-sweetened beverages, desserts and fried foods is associated with an increased risk of T2DM [44, 45]. However, whether or not the majority of the study participants utilised a particular food was unverified and therefore, a validated food frequency questionnaire would also have been useful.

Despite the increasing use of anti-hypertensives, BP control was suboptimal in our study population. With an attrition rate of nearly 40%, only 52 T2DM participants who took anti-hypertensive medications were able to maintain a target BP (both SBP and DBP) at follow up (Table 7). Majority were unable to achieve a desired target although they took more than one antihypertensive drug. This is disturbing given that high BP is by far the most critical risk factor for cardiovascular disease (CVD) and stroke [46]. Other studies that have explored the role of intensive BP control in preventing CVD have produced conflicting results. One study showed that a DBP of ≤80 mmHg could reduce the risk of CVD by 50% [47]. However, another study reported that SBP ≤120 mmHg was not associated with a reduced risk for CVD [48]. Notwithstanding this, our findings agree with several other studies that BP is poorly controlled among T2DM patients worldwide [49, 50].

Statins are well-known lipid lowering medications and the common one used by participants in this study is atorvastatin. More than half of the participants taking atorvastatin had good lipid profiles and this is consistent with the findings by Wong et al. [39]. Moreover, our study showed that there was a significant improvement in several lipid markers such as TG, LDL-c, HDL-c and VDL-c at follow up (Table 6). Whether atorvastatin interfered with glucose homeostasis is yet to be determined but our study confirms that atorvastatin is a potent drug for treating dyslipidaemia.

The present study does have some limitations. Firstly, because it was an observational longitudinal study, it was limited by confounding factors such as differences in dosage regimen. Dosage regimen refers to the modality of drug administration/doses per unit of time to reach a therapeutic objective. This comprises the time or frequency when the drug should be administered, the time between doses and the amount or unit dose of medicine to be administered at a specific time [51–53]. However, given the number of participants, each with a different medication dosage at a point in time, it was difficult to take into consideration the dosage regimen. At the same time, certain tests especially FBG are influenced by biological variation even when fluoride tubes are used. For example, stressful situations in the hours preceding FBG test could increase FBG levels [54]. Thus, we were unable to provide a full explanation on the poor drug response among some participants. Secondly, a clinical randomised control trial would have eliminated potential confounding factors, and also shed further light on the effect of the various medications in lowering modifiable risk factors. Thirdly, the sample size of the study was small and therefore cannot be representative of the entire T2DM population. Finally, over 40% of the participants were lost to follow up and this may have had an effect on our assessments.

## Conclusion

Our study showed that the use of statins is effective for improving lipid profiles and can be regarded as a potent medication for treating dyslipidaemia. However, utilisation of oral hypoglycaemic agents whether as a monotherapy, combination or polytherapy was not effective for achieving plasma glucose targets of <7%. This is alarming and therefore, alternative approaches including a less sedentary lifestyle while engaging in vigorous exercise may reduce weight and obesity; enforcing healthy eating practices and administration of single/fixed-dose combination tablets or pills with minimal side effects may improve medication adherence (Additional file 1: Table S1).

## Additional file

**Additional file 1: Table S1.** Morisky adherence scale-8 (MMAS-8).

## Abbreviations

BIG: biguanide
SUA: sulfonylurea
TNZ: thiazolidinedione
ACEI: angiotensin converting enzyme inhibitor
CCB: calcium channel blocker
ARB: angiotensin II receptor blocker
CAD: central acting drug
T2DM: type II diabetes mellitus
