# Supplementary material for: Profiling of cardio-metabolic risk factors and medication utilisation among Type II diabetes patients in Ghana: a prospective cohort study
Source: Clin Transl Med. 2017 Sep 7;6:32. doi: 10.1186/s40169-017-0162-5 (PMC5587509; doi:10.1186/s40169-017-0162-5)
Supplement: Supplementary file 1 — Additional file 1: Table S1. Morisky adherence scale-8 (MMAS-8). [file 40169_2017_162_MOESM1_ESM.docx]

|  |  | Drug Adherence status | |
| --- | --- | --- | --- |
| Variables | Total | Yes | No |
| Do you sometimes forget to take your medicine? | 239 | 104(43.2) | 135(56.0) |
|  |  |  |  |
| People sometimes miss taking their medicines for reasons other than forgetting | 239 | 71(29.5) | 168(69.7) |
|  |  |  |  |
| Have you ever cut back or stopped taking your medicine without telling your doctor? | 239 | 62(25.7) | 177(73.4) |
|  |  |  |  |
| When you travel or leave home, do you sometimes forget to bring along your medicine? | 239 | 108(44.8) | 131(54.4) |
|  |  |  |  |
| Did you take your medicines yesterday? | 239 | 219(90.9) | 20(8.3) |
|  |  |  |  |
| When you feel like your symptoms are under control, do you sometimes stop taking your medicine? | 239 | 48(19.9) | 191(79.3) |
|  |  |  |  |
| Taking medicine every day is a real inconvenience for some people. Do you ever feel hassled about sticking to your treatment plan? | 239 | 40(16.6) | 199(82.6) |

**Table: S1 Morisky adherence scale-8 (MMAS-8).**

Comparatively, a higher proportion of 135 individuals (56.0%) did not forget to take their medication; 168 (69.7%) missed their medications for reasons other than forgetting e.g. side effects; 177 (73.4%) did not cut back taking their medication without the knowledge of their health practitioner; 108 (44.8%) travelled along with their medications; 191 (79.3%) took their medicines while their symptoms subsided and 199 (82.6%) did not feel pressured about adhering to their dosage regimen.
